# Supplementary material for: Comparing molnupiravir and nirmatrelvir/ritonavir efficacy and the effects on SARS-CoV-2 transmission in animal models
Source: Nat Commun. 2023 Aug 7;14:4731. doi: 10.1038/s41467-023-40556-8 (PMC10406822; doi:10.1038/s41467-023-40556-8)
Supplement: Supplementary file 1 — Supplementary Information [file 41467_2023_40556_MOESM1_ESM.pdf]

Comparing molnupiravir and nirmatrelvir/ritonavir efficacy and the effects on SARS-CoV-2 transmission in animal models

Robert M Cox[1#], Carolin M Lieber[1#], Josef D Wolf[1], Amirhossein Karimi[1], Nicole A P Lieberman[2], Zachary M Sticher[3], Pavitra Roychoudhury[2], Meghan K Andrews[3], Rebecca E Krueger[3], Michael G Natchus[3], George R Painter[3,4], Alexander A Kolykhalov[3], Alexander L Greninger[2], Richard K Plemper[1*]

Supplementary Information

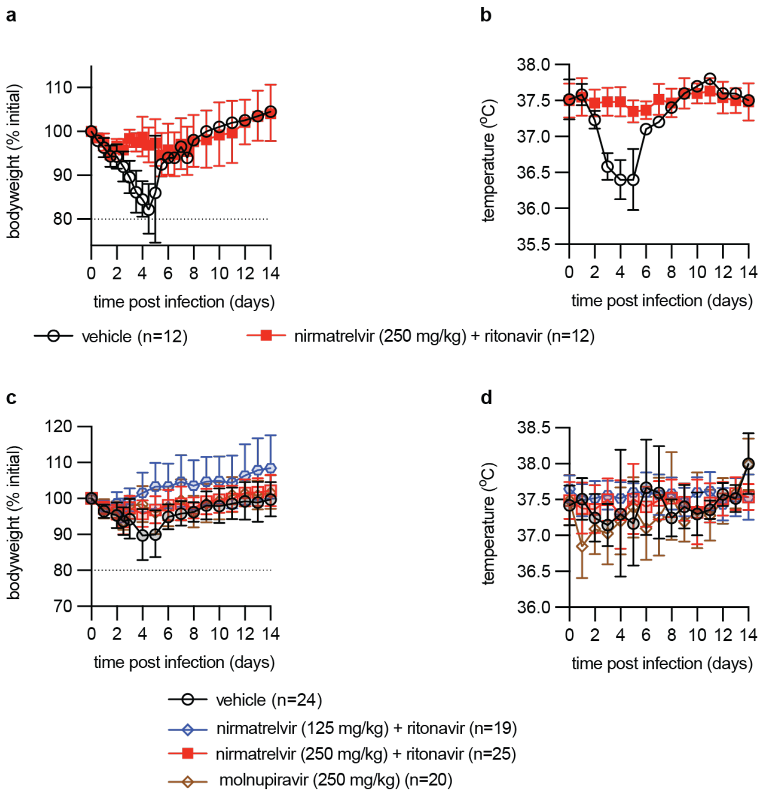

Supplementary Figure 1. Clinical signs in Roborovski dwarf hamsters. a-b, Bodyweight (a) and temperature (b) of VOC delta infected dwarf hamsters. **c-d**, Bodyweight (c) and temperature (d) of VOC omicron infected dwarf hamsters. The number of animals (n = numbers of independent animals) used in each experiment is shown. Symbols represent, and lines intersect, group means ± SD; dotted lines (a,c) denote predefined humane endpoint. Source data are provided as a Source Data file.

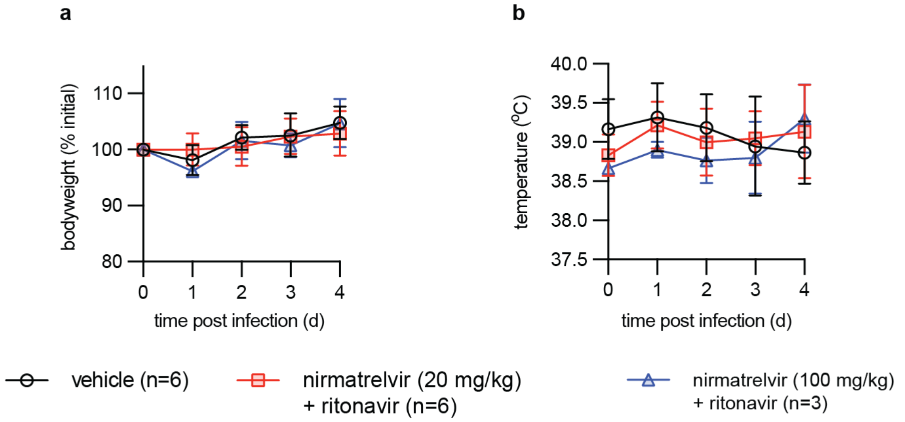

Supplementary Figure 2. Clinical signs of SARS-CoV-2 infected ferrets in efficacy study. a-b, Bodyweight (a) and temperature (b) of SARS-CoV-2 infected ferrets from figure 4. The number of animals (n = numbers of independent animals) used in each experiment is shown. Symbols represent group medians, lines intersect medians, and error bars represent 95% CIs. Source data are provided as a Source Data file.

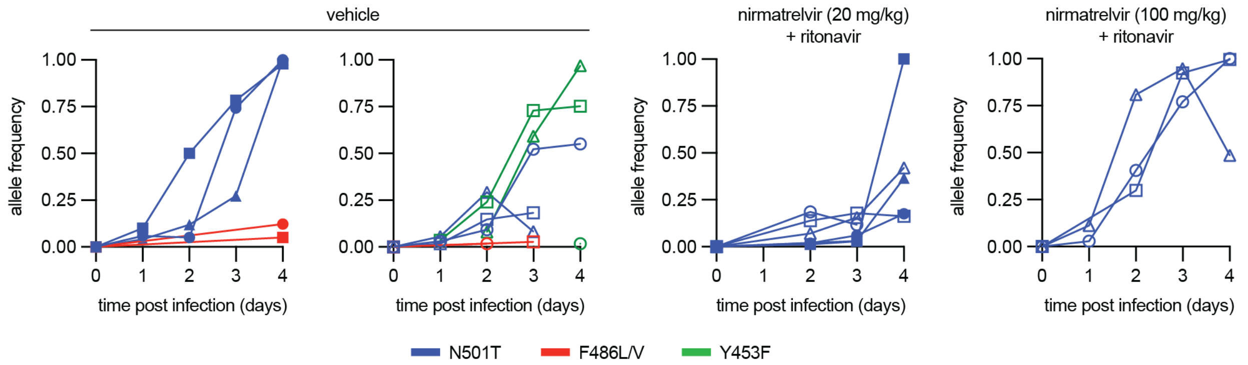

Supplementary Figure 3. Genetic variations in virus populations recovered from ferrets in the nirmatrelvir/ritonavir efficacy study. Relative allele frequency of all mutations that emerged in the spike protein of SARS-CoV-2 populations extracted from infected ferrets shown in Fig. 4c,e. Whole genome sequencing results are shown for animals treated with vehicle, 20 mg/kg, or 100 mg/kg nirmatrelvir/ritonavir as specified. Viral RNA was isolated from nasal lavage samples (study days 1-3) or extracted from nasal turbinates (study day 4). Symbols denote individual animals, line colors specify mutation in spike protein (N501T, Y453F, and F486L/V) that are characteristic for SARS-CoV-2 adaptation to mustelids; lines connect relative allele frequencies for independent biological repeats (individual animals).

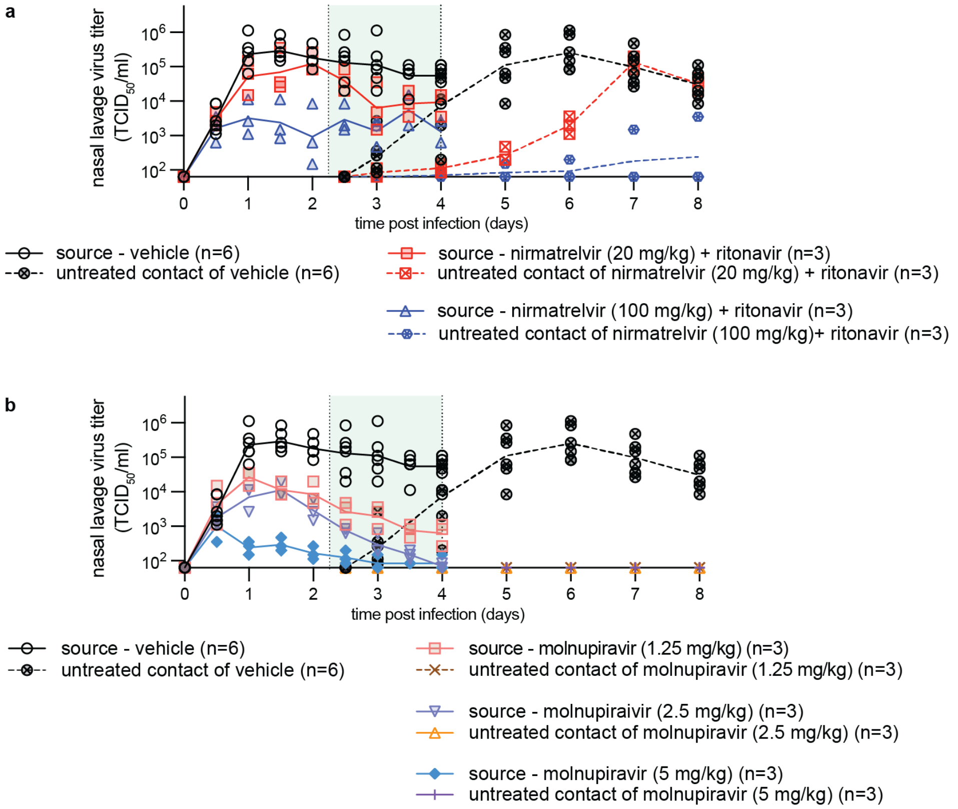

Supplementary Figure 4. Pharmacological intervention with SARS-CoV-2 transmission. a-b, Infectious titers in nasal lavages of SARS-CoV-2-infected ferrets treated with nirmatrelvir/ritonavir (a) or molnupiravir (b) as outlined in Fig. 5a. Results are shown for lavages taken from source animals in 12-hour intervals. Vehicle source and contact pairs apply to both a-b. The number of animals (n = numbers of independent animals) used in each experiment is shown. Symbols (a-b) represent individual biological repeats (individual animals); lines intersect geometric means; x-axes denote level of detection. Source data are provided as a Source Data file.

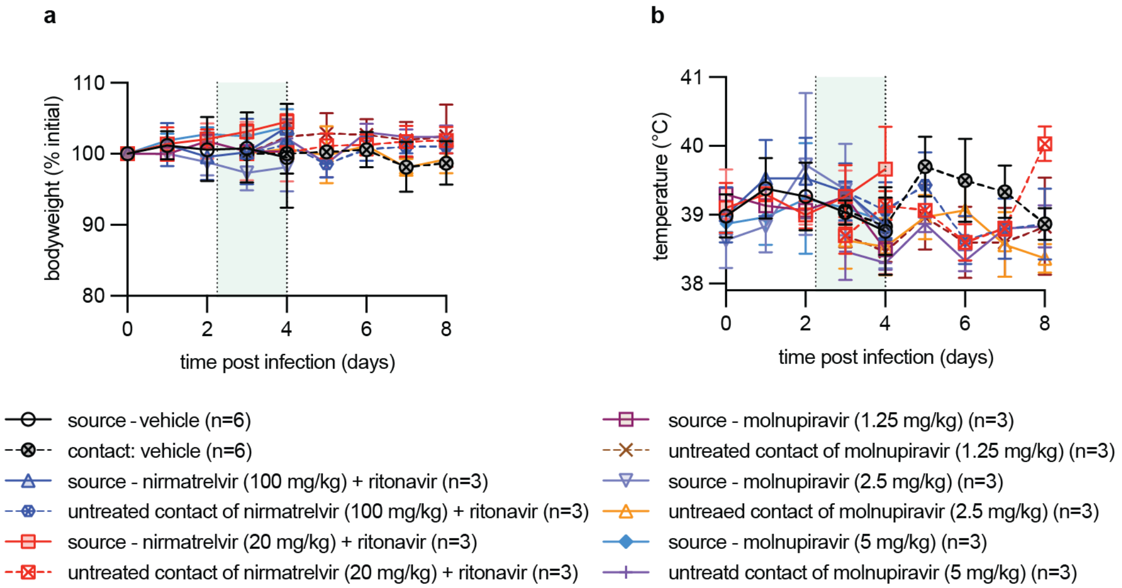

Supplementary Figure 5. Clinical signs of SARS-CoV-2 infected ferrets in the transmission study. a-b, Body weight (a) and temperature (b) of SARS-CoV-2 infected ferrets from figure 5. The number of animals (n = numbers of independent animals) used in each experiment is shown. Symbols represent group means ± SD; lines intersect means. Dashed vertical lines represent the time period of co-housing of source and naïve contact ferrets. Source data are provided as a Source Data file.

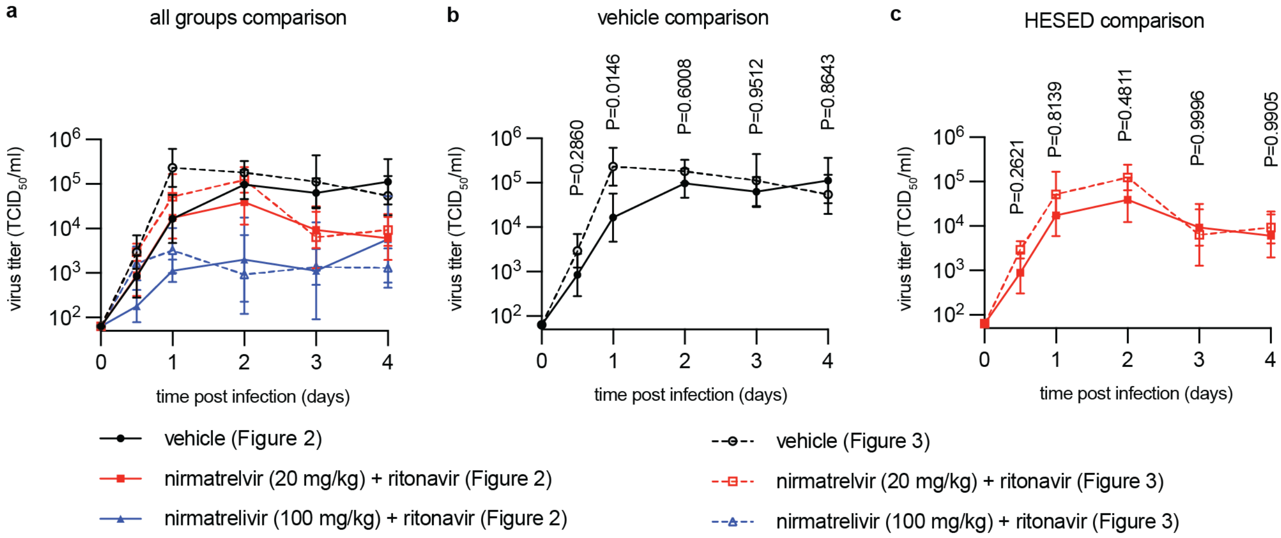

Supplementary Figure 6. Cross-study comparison of nirmatrelvir/ritonavir efficacy in ferrets. a-c, Virus titers in ferret nasal lavages of vehicle or nirmatrelvir/ritonavir-treated animals from studies presented in Fig. 4 (solid lines) and Fig. 5 (dashed lines). Shown are an overview of vehicle and all nirmatrelvir/ritonavir dose groups from both studies (a), vehicle-treated animals (b), and nirmatrelvir (20mg/kg) + ritonavir-treated animals (c). Symbols represent, and lines connect, geometric means ± SD. 2-way ANOVA with Sidak's post-hoc multiple comparison test (b-c); P values are shown. Source data are provided as a Source Data file.

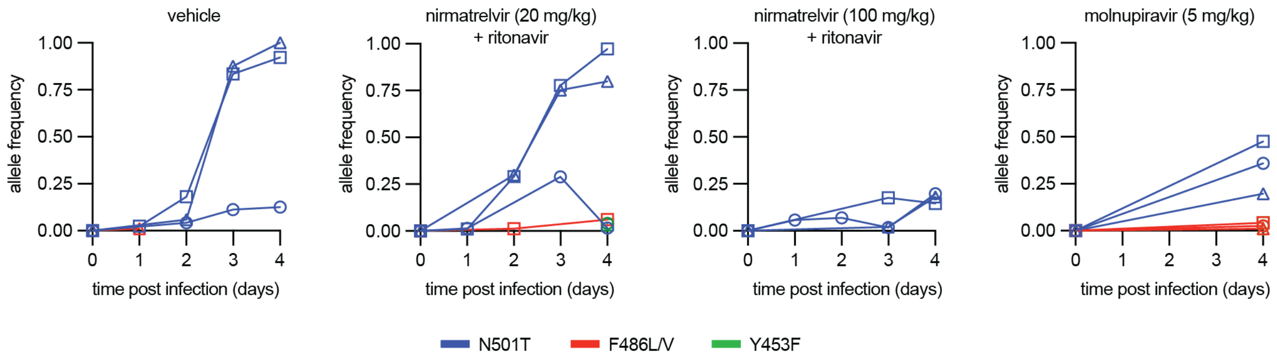

Supplementary Figure 7. Genetic variations in virus populations recovered from ferrets in the molnupiravir and nirmatrelvir/ritonavir transmission studies. Relative allele frequency of all mutations that

emerged in the spike protein of SARS-CoV-2 populations extracted from infected ferrets shown in Fig. 5b-c,f-g. Whole genome sequencing results are shown for animals treated with vehicle, 20 mg/kg or 100 mg/kg nirmatrelvir/ritonavir, or 5 mg/kg molnupiravir as specified. Viral RNA was isolated from nasal lavage samples (study days 1-3) or extracted from nasal turbinates (study day 4). Symbols denote individual animals, line colors specify mutation in spike protein (N501T, Y453F, and F486L/V) that are characteristic for SARS-CoV-2 adaptation to mustelids; lines connect relative allele frequencies for independent biological repeats (individual animals).

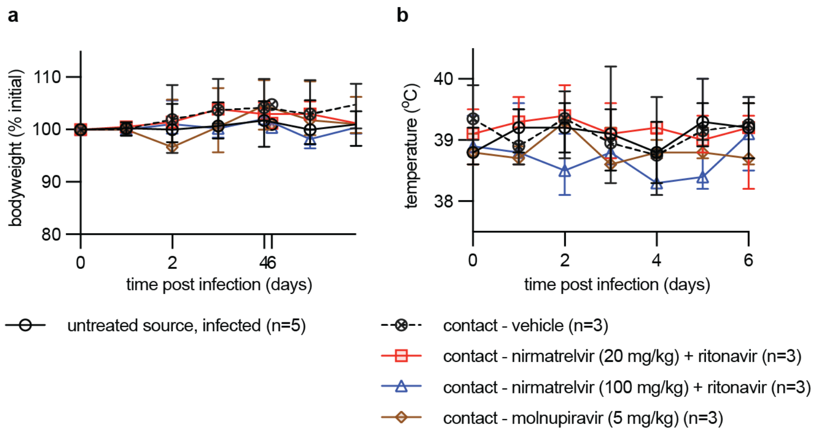

Supplementary Figure 8. Clinical signs of SARS-CoV-2 infected ferrets in the prophylactic treatment study. a-b, Body weight (a) and temperature (b) of SARS-CoV-2 infected ferrets from figure 6. The number of animals (n = numbers of independent animals) used in each experiment is shown. Symbols represent group means ± SD; lines intersect means. Infected source and prophylactically treated contact ferrets were co-housed starting 12 hours after infection. Source data are provided as a Source Data file.
