# Supplementary material for: Comparing molnupiravir and nirmatrelvir/ritonavir efficacy and the effects on SARS-CoV-2 transmission in animal models
Source: Nat Commun. 2023 Aug 7;14:4731. doi: 10.1038/s41467-023-40556-8 (PMC10406822; doi:10.1038/s41467-023-40556-8)
Supplement: Supplementary file 3 — Description of Additional Supplementary Files [file 41467_2023_40556_MOESM3_ESM.pdf]

Comparing molnupiravir and nirmatrelvir/ritonavir efficacy and the effects on SARS-CoV-2 transmission in animal models

Robert M Cox[1#], Carolin M Lieber[1#], Josef D Wolf[1], Amirhossein Karimi[1], Nicole A P Lieberman[2], Zachary M Sticher[3], Pavitra Roychoudhury[2], Meghan K Andrews[3], Rebecca E Krueger[3], Michael G Natchus[3], George R Painter[3,4], Alexander A Kolykhalov[3], Alexander L Greninger[2], Richard K Plemper[1*]

Description of Additional Supplementary Files

1) File Name: Supplementary Data 1.
   Description: All statistical analyses

2) File Name: Supplementary Data 2.
   Description: Mass spectrometry raw data of PK studies

3) File name: Source Data.
   Description: All quantitative raw data
