# Supplementary material for: Comparing molnupiravir and nirmatrelvir/ritonavir efficacy and the effects on SARS-CoV-2 transmission in animal models
Source: Nat Commun. 2023 Aug 7;14:4731. doi: 10.1038/s41467-023-40556-8 (PMC10406822; doi:10.1038/s41467-023-40556-8)
Supplement: Supplementary file 6 — Reporting Summary [file 41467_2023_40556_MOESM6_ESM.pdf]

nature portfolio

Corresponding author(s): Richard K Plemper, Ph.D.

Last updated by author(s): Jun 27, 2023

Reporting Summary

Nature Portfolio wishes to improve the reproducibility of the work that we publish. This form provides structure for consistency and transparency in reporting. For further information on Nature Portfolio policies, see our Editorial Policies and the Editorial Policy Checklist.

Statistics

For all statistical analyses, confirm that the following items are present in the figure legend, table legend, main text, or Methods section.

n/a	Confirmed	
☐	☒	The exact sample size (*n*) for each experimental group/condition, given as a discrete number and unit of measurement
☐	☒	A statement on whether measurements were taken from distinct samples or whether the same sample was measured repeatedly
☐	☒	The statistical test(s) used AND whether they are one- or two-sided<br>*Only common tests should be described solely by name; describe more complex techniques in the Methods section.*
☒	☐	A description of all covariates tested
☐	☒	A description of any assumptions or corrections, such as tests of normality and adjustment for multiple comparisons
☐	☒	A full description of the statistical parameters including central tendency (e.g. means) or other basic estimates (e.g. regression coefficient) AND variation (e.g. standard deviation) or associated estimates of uncertainty (e.g. confidence intervals)
☐	☒	For null hypothesis testing, the test statistic (e.g. *F*, *t*, *r*) with confidence intervals, effect sizes, degrees of freedom and *P* value noted<br>*Give P values as exact values whenever suitable.*
☒	☐	For Bayesian analysis, information on the choice of priors and Markov chain Monte Carlo settings
☒	☐	For hierarchical and complex designs, identification of the appropriate level for tests and full reporting of outcomes
☒	☐	Estimates of effect sizes (e.g. Cohen's *d*, Pearson's *r*), indicating how they were calculated

Our web collection on statistics for biologists contains articles on many of the points above.

Software and code

Policy information about availability of computer code

Data collection	Excel (Versions 16.52; Microsoft) and Numbers (version 10.1; Apple) used for most data collection. Reverse transcription qPCR data was collected using an Applied Biosystems StepOnePlus PCR system using the StepOnePlus software (Version 2.1; Applied Biosystems). SARS-CoV-2 positive specimens were sequenced using IDT xGen SARS-CoV-2 Amplicon Panel (previously Swift Biosciences SNAP panel).
Data analysis	Statistical analyses were performed in the Prism (GraphPad) software package (version 9.4.1). Reverse transcription RT-qPCR data were collected and analyzed using the QuantStudio Design and Analysis (version 1.5.2; Applied Biosystems) software package. Figures were composed using Adobe Illustrator (version 27.5). PK models were generated with Phoenix WinNonLin 8.3.3.33 (Certara) software package. Reverse transcription qPCR data was analyzed using the StepOnePlus software (Version 2.1; Applied Biosystems). SARS-CoV-2 next-generation sequencing analysis was performed using using TAYLOR default settings available at https://github.com/greninger-lab/covid_swift_pipeline. Four-parameter variable slope regression modeling and statistical analyses were performed in the Prism (GraphPad) software package. (Version 9.4.1).

For manuscripts utilizing custom algorithms or software that are central to the research but not yet described in published literature, software must be made available to editors and reviewers. We strongly encourage code deposition in a community repository (e.g. GitHub). See the Nature Portfolio guidelines for submitting code & software for further information.

Data

Policy information about availability of data

All manuscripts must include a data availability statement. This statement should provide the following information, where applicable:

- Accession codes, unique identifiers, or web links for publicly available datasets
- A description of any restrictions on data availability
- For clinical datasets or third party data, please ensure that the statement adheres to our policy

The metagenomic sequencing reads generated in this study have been deposited in the NCBI BioProject database under accession code PRJNA894555 (https://www.ncbi.nlm.nih.gov/bioproject/?term=PRJNA894555). All other data generated in this study are provided in this published article and the Supplementary Information/Source Data file.

Human research participants

Policy information about studies involving human research participants and Sex and Gender in Research.

Reporting on sex and gender	This study did not involve human research participants.
Population characteristics	N/A
Recruitment	N/A
Ethics oversight	N/A

Note that full information on the approval of the study protocol must also be provided in the manuscript.

Field-specific reporting

Please select the one below that is the best fit for your research. If you are not sure, read the appropriate sections before making your selection.

[x] Life sciences   [ ] Behavioural & social sciences   [ ] Ecological, evolutionary & environmental sciences

For a reference copy of the document with all sections, see nature.com/documents/nr-reporting-summary-flat.pdf

Life sciences study design

All studies must disclose on these points even when the disclosure is negative.

Sample size	Appropriate sample sizes were determined using the Resource Equation and Power analysis. Unless otherwise noted, at least three samples were used for each group.
Data exclusions	No data was excluded from this manuscript.
Replication	All experimental data were reliably produced; the number of independent biological repeats and, when applicable, technical repeats is specified for each experiment in the figure legends. All experiments yielding quantitative raw data were independently repeated at least 3 times.
Randomization	Sourced animals were randomly assigned to cages by animal support staff blinded for the study. After a resting period of at least 72 hours, cages were randomly assigned to study groups, animals weighed, and infected as specified in figure legends.
Blinding	Animal support staff blinded for the study assigned incoming animals randomly to cages. The investigators were not blinded to group allocation for data collection and analysis for any experiment performed in this study due to size of the research group with clearance for experimentation under high biocontainment conditions required for work with live SARS-CoV-2. Ferrets and dwarf hamsters cannot be handled by a single investigator under high biocontainment and resources available did not allow involvement of additional personnel that would have been required for blinding.

Reporting for specific materials, systems and methods

We require information from authors about some types of materials, experimental systems and methods used in many studies. Here, indicate whether each material, system or method listed is relevant to your study. If you are not sure if a list item applies to your research, read the appropriate section before selecting a response.

Materials & experimental systems

n/a	Involved in the study
☒	☐ Antibodies
☐	☒ Eukaryotic cell lines
☒	☐ Palaeontology and archaeology
☐	☒ Animals and other organisms
☒	☐ Clinical data
☒	☐ Dual use research of concern

Methods

n/a	Involved in the study
☒	☐ ChIP-seq
☒	☐ Flow cytometry
☒	☐ MRI-based neuroimaging

Eukaryotic cell lines

Policy information about cell lines and Sex and Gender in Research

Cell line source(s)	African green monkey kidney cells VeroE6 stably expressing TMPRSS2 (VeroE6-TMPRSS2) (BPS Bioscience #78081)<br>human lung adenocarcinoma epithelial cells Calu-3 (ATCC® HTB-55™)
Authentication	Cells were authenticated by the supplier or by morphological appearance and susceptibility to virus infection including SARS-CoV-2.
Mycoplasma contamination	Cell lines were confirmed mycoplasma-negative when obtained from the supplier, followed by preparation and cryo-preservation of master and working stocks. Individual working stocks were replaced every three months. All cell lines in use in the laboratory were routinely retested for mycoplasma contamination in 3-months intervals and tested negative.
Commonly misidentified lines<br>(See ICLAC register)	No commonly misidentified cell lines were used in this study.

Animals and other research organisms

Policy information about studies involving animals; ARRIVE guidelines recommended for reporting animal research, and Sex and Gender in Research

Laboratory animals	This study used female ferrets (Mustela putorius furo), family mustelids, genus mustela, 6-10 months of age and male and female Roborovski dwarf hamsters (Phodopus roborovskii), family cricetidae, genus phodopus, 3-4 months of age.
Wild animals	This study did not involve wild animals.
Reporting on sex	Biological sex of research animals was determined based on examination of external genitalia. Stated in the abstract that ferret studies were carried out with female animals only. Per our IACUC protocol, co-housing studies may not be carried out with male ferrets, since males are extremely territorial and highly combative when co-housed, resulting in severe injury from fight wounds that would require termination of the study. Roborovski dwarf hamster studies were carried out with approximately equal numbers of male and female animals, randomly assigned to individual study groups, but study groups were not statistically powered to assess sex as a biological variable. Since no signs of biological sex-dependency of study outcome were noted in the randomly assemble groups, group sizes were not further expanded. Disintegrated quantitative results for male and female dwarf hamsters are provided in the source data file; overall, this study used 87 male dwarf hamster, 95 female dwarf hamsters (182 dwarf hamsters total) and 95 female ferrets.
Field-collected samples	This study did not involve field-collected samples.
Ethics oversight	Experiments with SARS-CoV-2 involving ferrets were approved by the Georgia State Institutional Animal Care and Use Committee under protocol A20031. Experiments with SARS-CoV-2 involving Roborovski dwarf hamsters were approved by the Georgia State Institutional Animal Care and Use Committee under protocol A21019. All experiments using infectious SARS-CoV-2 strains were approved by the Georgia State Institutional Biosafety Committee under protocol B20016 and performed in BSL-3/ABSL-3 facilities at the Georgia State University.

Note that full information on the approval of the study protocol must also be provided in the manuscript.
